# Trends in educational inequalities in obesity in 15 European countries between 1990 and 2010

**DOI:** 10.1186/s12966-017-0517-8

**Published:** 2017-05-08

**Authors:** Kristina Hoffmann, Rianne De Gelder, Yannan Hu, Matthias Bopp, Jozsef Vitrai, Eero Lahelma, Gwenn Menvielle, Paula Santana, Enrique Regidor, Ola Ekholm, Johan P. Mackenbach, Frank J. van Lenthe

**Affiliations:** 1 Department of Public Health, Erasmus MC, University Medical Center Rotterdam, P.O. Box 2040, 3000 CA Rotterdam, Netherlands; 2 Mannheim Institute of Public Health, Social and Preventive Medicine, Medical Faculty Mannheim, Heidelberg University, Mannheim, Germany; 3 Epidemiology, Biostatistics and Prevention Institute, University of Zürich, Zürich, Switzerland; 4 National Institute for Health Development, Budapest, Hungary; 5 Department of Public Health, University of Helsinki, Helsinki, Finland; 6 Sorbonne Universités, INSERM, Institut Pierre Louis d’Epidémiologie et de Santé Publique (IPLESP UMRS 1136), Paris, France; 7 Departamento de Geografia, Centro de Estudos de Geografia e de Ordenamento do Territorio (CEGOT), Colégio de S. Jerónimo, Universidade de Coimbra, Coimbra, Portugal; 8 Department of Preventive Medicine and Public Health, Universidad Complutense de Madrid, Madrid, Spain; 9 National Institute of Public Health, University of Southern Denmark, Copenhagen, Denmark

**Keywords:** Health inequalities, Obesity, Time trends

## Abstract

**Background:**

The prevalence of obesity increased dramatically in many European countries in the past decades. Whether the increase occurred to the same extent in all socioeconomic groups is less known. We systematically assessed and compared the trends in educational inequalities in obesity in 15 different European countries between 1990 and 2010.

**Methods:**

Nationally representative survey data from 15 European countries were harmonized and used in a meta-regression of trends in prevalence and educational inequalities in obesity between 1990 and 2010. Educational inequalities were estimated by means of absolute rate differences and relative rate ratios in men and women aged 30–64 years.

**Results:**

A statistically significant increase in the prevalence of obesity was found for all countries, except for Ireland (among men) and for France, Hungary, Italy and Poland (among women). Meta-regressions showed a statistically significant overall increase in absolute inequalities of 0.11% points [95% CI 0.03, 0.20] per year among men and 0.12% points [95% CI 0.04, 0.20] per year among women. Relative inequalities did not significantly change over time in most countries. A significant reduction of relative inequalities was found among Austrian and Italian women.

**Conclusion:**

The increase in the overall prevalence aligned with a widening of absolute but not of relative inequalities in obesity in many European countries over the past two decades. Our findings urge for a further understanding of the drivers of the increase in obesity in lower education groups particularly, and an equity perspective in population-based obesity prevention strategies.

**Electronic supplementary material:**

The online version of this article (doi:10.1186/s12966-017-0517-8) contains supplementary material, which is available to authorized users.

## Background

Socioeconomic inequalities in mortality and self-reported morbidity have been extensively documented in national and international studies [1–7]. For both of these outcomes, and either defined by educational or income level, rates are higher in the lower socioeconomic groups. Patterns of inequality are, however, not static: a larger absolute decline in mortality rates in lower as compared to higher socioeconomic groups, has resulted in a narrowing of the difference in mortality rates between low and high socioeconomic groups (i.e., a decline in absolute inequalities) in many European countries over the past two decades. At the same time, the larger decline in the low as compared to the high socioeconomic group increased the ratio of mortality in the lower as compared to the higher socioeconomic group (i.e., an increase in relative inequality) [8]. Thus, it is important to consider both absolute and relative measures of inequality in studies on trends in health inequalities [9].

A proper understanding of these trends requires an analysis of trends in inequalities in major determinants of mortality [5]. Smoking is often mentioned as the single most important mediating factor of inequalities in mortality. In line with the above-mentioned findings, we recently found a decline in absolute inequalities in smoking-attributable mortality, among men [10]. At the same time however, countries witnessed a substantial increase in obesity, and current socioeconomic inequalities in obesity across Europe suggest that the increase was larger among lower socioeconomic groups [11]. Elimination of socioeconomic inequalities in obesity might reduce inequalities in both mortality and morbidity substantially [12]; a widening of inequalities in obesity however, would buffer the impact of a decline of smoking attributable mortality on socioeconomic inequalities in mortality [13].

To better understand trends in socioeconomic inequalities in mortality, as well as to formulate hypotheses that help to identify the underlying causes of socioeconomic inequalities in obesity, it is important to compare trends in obesity-related disparities between countries [14]. Previous studies, mostly among residents of single nations, showed persistent or increasing socioeconomic inequalities in obesity in different countries [15–22]. One of the few international cross-country comparative trend studies, including 4 European countries, reported generally persistent social inequalities in obesity [23]. Studies on long-term changes in the prevalence of and inequalities in obesity across many European countries based on harmonized data sets are still scarce.

Acknowledging that Europe is a highly diverse world region known for health inequalities, the EU-funded “Developing methodologies to reduce inequalities in the determinants of health” project (DEMETRIQ) aimed to construct a harmonized dataset of health outcomes by indicators of socioeconomic position over the last decades [24]. The study offered the unique possibility to systematically assess the trends in socioeconomic inequalities in obesity in 15 European countries between 1990 and 2010.

## Methods

### Data sources

We obtained nationally representative health surveys from 15 European countries with more than two surveys available between the time period 1990 and 2010. Data came from the same survey over time for most of the countries, except for Austria, France, Hungary, and Italy (Table 1). The different data sources within these four countries have a high comparability [25–31], and thus could be included to analyze the trends over time. The age range used for all countries in the analysis was 30–64 years. In Polish data information on age was provided in 10-year groups and thus for Poland the upper limit of 69 was used instead. Older respondents were excluded because the upper age limits differed between countries, which could have influenced the comparability of the level of inequalities in obesity. Younger respondents were excluded because many of them were still receiving full-time education and thus most likely represent a selective group with higher education. The number of included respondents per year ranged from 2238 (Finland in 1993) to 67,485 (Italy in 2005). For trends in education related inequalities, we included 15 countries with 60 country-year observations. To test the robustness of education as an indicator of socioeconomic position we performed sensitivity analysis analyzing inequality trends by occupational class, including 14 countries with 57 country-year observations.

**Table 1:** List of countries and corresponding sources of data included in the analyses

Country	Survey year	Survey name	Age range	Number of respondents (per year)	Assessment of weight and height
Austria	1991/1999	Micro Census	30–64	21,867 ~ 22,557	Self-reported
	2006	Health Interview Survey	30–64	8776	Self-reported
Belgium	1997/2001/2004/2008	Health Interview Survey	30–64	4874 ~ 5928	Self-reported
Denmark	1994/2000/2005/2010	Danish Health and Morbidity Survey	30–64	2668 ~ 10,120	Self-reported
Finland	1993/1995/1997/1999/2001/2003/2005/2007/2009	Health Behaviour and Health	30–64	2238 ~ 2599	Self-reported
France	1991–1992	Enquête Décennale Santé	30–64	9619	Self-reported
	2000/2005/2010	Baromètre santé	30–64	8108 ~ 17,281	Self-reported
Hungary	1994/2000/2003	National Health Interview Survey	30–64	2997 ~ 4153	Self-reported
	2009	European Health Interview Survey	30–64	3012	Self-reported
Ireland	1998/2002/2007	Survey of Lifestyle and Nutrition	30–64	3651 ~ 6488	Measured
Italy	1994/2000/2005	Health and Health Care Utilization	30–64	29,274 ~ 67,485	Self-reported
	1990/2010	Multipurpose Family Survey. Aspects of Daily Living	30–64	24,190 ~ 31,318	Self-reported
Netherlands	1997/2000/2005/2009	Permanent Survey on Living Conditions (POLS)	30–64	4064 ~ 5118	Self-reported
Norway	1998/2002/2005/2008	Norwegian Level of living surveys	30–64	3990 ~ 4205	Self-reported
Poland	1996/2004/2009	Polish Health Interview Survey	30–69	21,353 ~ 29,712	Self-reported
Portugal	1995–1996/1998–1999/2005–2006	National Health Survey	30–64	19,166 ~ 22,574	Self-reported
Scotland	1995/1998/2003	Scottish Health Survey	30–64	5036 ~ 5997	Measured
Spain	1993/2001/2006	National Health Survey	30–64	11,229 ~ 17,602	Self-reported
Switzerland	1992/1997/2002/2007	Swiss Health Survey	30–64	6400 ~ 10,796	Self-reported

### Variables

To measure obesity, the body mass index (BMI) was calculated as weight in kilograms divided by square height in meters in all surveys. In 13 countries weight and height were self-reported, whereas in Ireland and Scotland weight and height were objectively measured. Obesity was defined as BMI ≥30 kg/m^2^ [32].

Socioeconomic position was measured by educational level. Education levels were recorded as the highest level of education completed or currently being attended by a person. They were harmonized on the basis of the International Standard Classification of Education (ISCED) [33] and reclassified into 3 categories: levels 0–2 (no, primary or lower secondary education, considered “low-educated”), levels 3–4 (upper secondary and post-secondary non-tertiary education, considered “middle-educated”), levels 5–6 (tertiary education, considered “high-educated”).

Occupational classes were categorized as “manual” (considered the lower level) versus “non-manual” (considered the higher level). Respondents who were not economically active, and who could not be classified on the basis of their last or main occupation were classified as missing. Farmers and self-employed were excluded from the analysis. Since results are generally similar to those obtained for education, they will be presented in an online supplementary file.

### Statistical methods

For each country, the prevalence rates of obesity were calculated by year, sex, and level of education, occupational class, and age-standardized to the European Standard Population using the direct standardization method [34]. For visual between-country comparison total prevalence rates and prevalence rates by socioeconomic group indicator were plotted as over time line charts.

Inequalities were measured by means of absolute prevalence rate differences (RD) and relative prevalence rate ratios (RR) of low versus high level of socioeconomic position. A bootstrap procedure with 1000 iterations was used to calculate 95% confidence intervals. Survey weights were available in some countries or years, but not in all. Thus, unweighted results are reported in the results section. A previous study based on the same data sources and with self-assessed health as the outcome found essentially similar results if weighted or unweigthed data were used [35].

To study the trends over time in each country and in the ensemble of countries as a whole, we employed meta-regression with random effects models, using the DerSimonian and Laird method [36]. The year of data collection was used as the only independent variable in the models and the total prevalence of obesity, the prevalence of obesity by level of socioeconomic position; the absolute (RD) and relative inequalities (RR) by socioeconomic position were included as the dependent variables. The country-specific regression parameters and their 95% confidence intervals were displayed as forest plots and meta-analyses were performed to calculate overall random effect estimates for all countries. Data were analyzed for males and females separately. All analyses were performed using Stata/SE 13.1 (StataCorp, Texas, US).

## Results

### Time trends in obesity prevalence

**Fig. 1:**
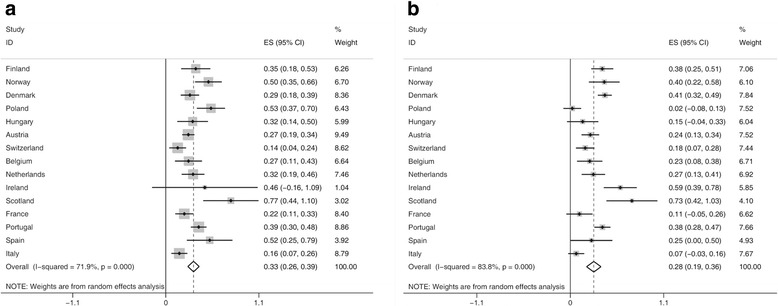
Forest plot of meta-regression slopes for trends in prevalence of total obesity (BMI ≥30 kg/m^2^) in (**a**) men and (**b**) women. ES, effect estimator (% points change of obesity prevalence per year); CI, confidence interval

The forest plots in Fig. 1a and b display the results of the meta-regression analysis for the trends in age-standardized obesity prevalence in 15 European countries between 1990 and 2010. An increase in the prevalence of obesity was found for almost all countries, but varied in magnitude between countries. In men, the pooled estimates for all countries indicated an increase of 0.33% points in the prevalence of obesity per year [95% CI 0.26, 0.39]. Only in Ireland this increase was not statistically significant. The increase in the prevalence of obesity was particularly large in Scotland, Norway and Poland. In Switzerland, France and Italy, the overall increase was lower than the average increase across all countries included. In women, the pooled estimates for all countries indicated an increase of 0.28% points per year [95% CI 0.19, 0.36] in the past two decades. In Poland, Hungary, France, Italy and Spain the increases were not statistically significant. Denmark, Scotland and Ireland witnessed an above average increase in the prevalence of obesity.

### Between-country comparison of changes in prevalence rates over time

**Fig. 2:**
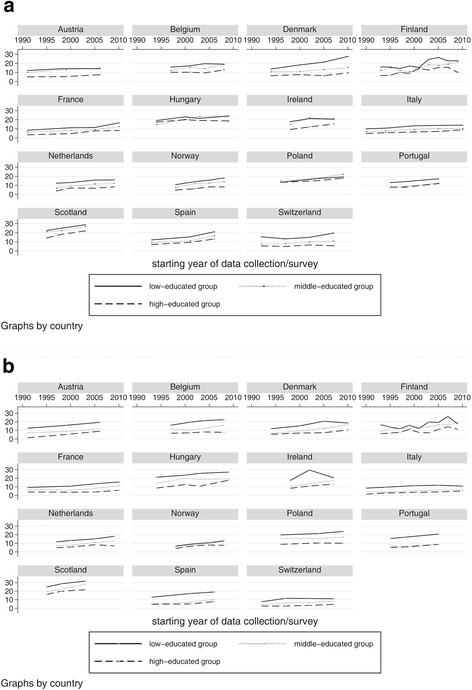
Prevalence (%) of obesity (BMI ≥30 kg/m^2^) over time in (**a**) men and (**b**) women aged 30–64 in all countries stratified by educational level (ISCED 0–2, ISCED 3–4, ISCED 5–6)

Figure 2 presents a graphical overview of the changes of obesity prevalence over time per country and by education. The prevalence rates in low, middle and high educational classes have increased over the past two decades in all 15 countries in both men (Fig. 2a) and women (Fig. 2b). Visual inspection of the graphs showed that obesity rates have been highest in low educated persons between 1990 and 2010, except in males in Hungary, Ireland and Poland (Fig. 2a). Even though the trends followed the same direction, the gaps between low and high educational groups differed in size and shape between the countries. Whereas in most of the countries the gaps in the prevalence of obesity between the educational levels seemed to remain relatively constant over time, a noticeable widening of the gap was particularly observed in Danish men (Fig. 2a).

### Time trends in absolute and relative inequalities in obesity prevalence

The pooled estimates for all countries by educational level revealed a larger increase in obesity prevalence in the low educational group (0.40% points per year [95% CI 0.30, 0.49]) as compared to the high educational group (0.29% points per year [95% CI 0.20, 0.39]) in men [see Additional file 1: Figure S1a, b]. Country-specific estimates for the low educated group showed an increase in all countries (although not statistically significant in Hungary, Austria, Switzerland and Ireland). Increases were found as well in men in the high educational groups (although not statistically significant in Denmark, Hungary, Austria, Switzerland and Belgium), but they were generally smaller. In Finland, the Netherlands and Portugal, the increases were relatively similar between men in the low and high educational groups. A smaller increase in obesity prevalence in the low as compared to the high educational group was found in men in Ireland and Scotland. In women, the pooled estimates for all countries by educational level showed a larger increase in obesity in the low (0.40% points per year [95% CI 0.32, 0.49]) as compared to the high educated group (0.25% points per year [95% CI 0.18, 0.33]). [see Additional file 1: Figure S1c–d]. Among women in the low educational group, an increase in the prevalence of obesity was also observed in all countries (although not statistically significant in Finland, Switzerland and Ireland). They were generally larger than the increases in women in the high educational group. A smaller increase in obesity prevalence in women in the low as compared to the high educational group was found in Finland, Hungary, Austria, Ireland and Italy.

**Fig. 3:**
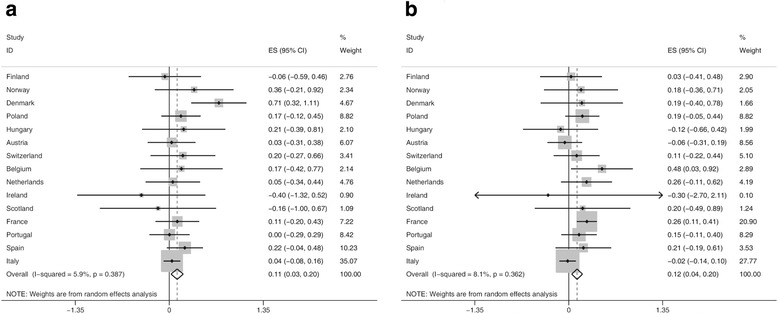
Forest plot of meta-regression slopes for trends in absolute inequality in obesity prevalence (BMI ≥30 kg/m^2^) in (**a**) men and (**b**) women. RD, rate difference between low and high educational level; ES, effect estimator (% points change of absolute inequalities in obesity per year); CI, confidence interval

Meta-regressions of absolute educational inequalities in obesity resulted in a statistically significant overall increase in absolute inequalities of: 0.11% points [95% CI 0.03, 0.20] per year in men and 0.12% points [95% CI 0.04, 0.20] per year in women. No statistically significant trend was observed in the majority of the individual countries (Fig. 3a, b). The country-specific increase in absolute inequalities in obesity was only significant in Denmark in men (Fig. 3a) and in Belgium and France in women (Fig. 3b).

**Fig. 4:**
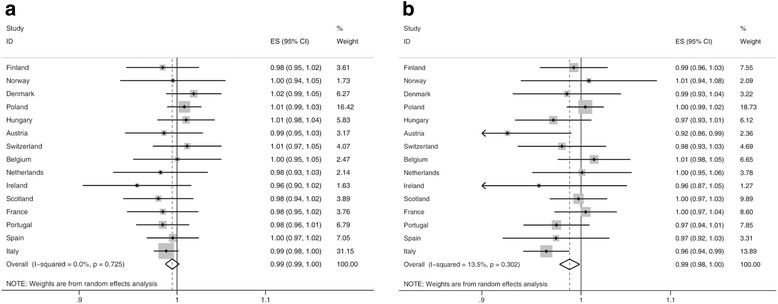
Forest plot of meta-regression slopes for trends in relative inequality in obesity prevalence (BMI ≥30 kg/m^2^) in (**a**) men and (**b**) women. RR, rate ratio between low and high educational level; ES, effect estimator (relative change of relative inequalities in obesity per year); CI, confidence interval

Figure 4a and b display the meta-regression results for the time trend of relative inequalities in obesity. The overall estimates pooled for all countries in men and women separately were slightly below 1 indicating that relative inequalities did not significantly change over time. In Austrian and Italian women a significant reduction of relative inequalities was found (Austria: 0.92 [95% CI 0.86, 0.99]; Italy: 0.96 [95% CI 0.94, 0.99]).

## Discussion

### Main findings

Pooled analyses of data for 15 European countries showed an increase in the prevalence of obesity between 1990 and 2010 in both men and women. Increases in the prevalence were generally larger for those in the low as compared the high educated group. As a result, the pooled analysis showed an increase in absolute inequalities in obesity. At the country-level, this was statistically significant in Denmark in men and in Belgium and France in women. Relative inequalities were rather constant or slightly decreased between 1990 and 2010.

### Strengths and limitations

To the best of our knowledge, this study is the largest international comparison of time trends in socioeconomic inequalities in obesity across Europe, in terms of the number of countries and years included. Meta-regression was used to systematically analyze trends in both absolute and relative inequalities in obesity. Efforts have been undertaken to overcome heterogeneity over time and between countries.

Measures of educational levels were harmonized by using the International Standard Classification of Education (ISCED) [33]. Whereas most countries used highest educational level completed, surveys in the Netherlands asked for the highest level of education completed or attended. As a result, this might have generated larger proportions of persons in the middle and highest educational groups in the Netherlands as compared to the other countries. To the extent that obese persons more often did not complete the attended degree, educational gradients in obesity may have been underestimated in the Netherlands as compared to the other countries.

No single indicator however, captures the construct of socioeconomic position entirely. Other studies showed similar patterns of inequalities in obesity, measured by occupational status, income and educational level [15, 37, 38]. We also repeated the analyses for all countries using occupational class as an indicator of socioeconomic position from the same data sets, except for Portugal where no data on occupational position were available [see Additional file 2: Figure S2–S5]. Prevalence and inequality trends showed essentially similar patterns, thereby strengthening our findings for educational inequalities.

To facilitate the comparison between inequalities by different socioeconomic indicators, we used the RD and RR of low versus high education. However, these results might have been driven by changes in the distribution of educational level over time. For that reason we decided to repeat the analysis with indicators of inequalities that do take into account underlying distributions. We used the slope index of inequality (SII) and the relative index of inequality (RII) as an indicator for absolute and relative inequality, respectively. The SII in our analysis reflects the absolute difference in BMI between the least and the most advantaged individual, the RII can be interpreted as the relative risk of obesity for the least advantaged as compared to the most advantaged individual, both now taking into account the educational distribution by applying regression techniques [39].

The results were essentially similar. There was no discrepancy in significance of country-specific estimates of absolute inequalities measured by the RD and SII (see Additional file 3: Figure S6a, b). Only among women in Poland, we now found a significant increase in relative inequality measured by RII (see Additional file 3: Figure S7a, b).

Thus, these additional results support our main findings, e.g. an increase in absolute inequalities and stable relative inequalities between 1990 and 2010.

A potential limitation of our study concerned the assessment of BMI. In most of countries participants provided self-reported height and weight whereas in Ireland and Scotland these variables were measured. The heterogeneous nature of BMI assessment could have introduced potential bias in our analyses. It has been shown previously that obese people tend to underestimate their weight [40, 41]. Whether there are differences in reporting between educational groups has not been clearly answered [42–44]. Nevertheless, Boström and Diderichsen found that reporting bias and its potential effect on inequalities in obesity are rather small due to the underestimation of BMI in all socioeconomic groups [45]. According to their findings, we might have underestimated inequalities in women and overestimated inequalities in men. Sensitivity meta-analysis of total obesity prevalence including only countries with self-reported BMI-assessment revealed no significant reduction of heterogeneity calculated by I^2^-statistics and no significant change in the estimates (data not shown).

Not having had access to the exact participation rates for each survey is another aspect of the data collection process, which might have affected our results in different ways. Firstly, response rates may have declined over time, and - to the extent the response rate was positively associated with levels of obesity – we may have underestimated increases in obesity over time. Secondly, participation rates may have differed between countries, and thirdly, participation may have differed between high and low socioeconomic groups. Particularly the latter may have affected the magnitude of inequalities.

Other data collection procedures may have had little impact on the analyses, to the extent that they did not change over time within countries.

It cannot be ruled out that our underlying assumption for the meta-analyses that trends in the outcome measures follow a linear pattern might not always hold true. However, this technique provides a systematic overview of the inequality trends in obesity within the last two decades in European countries. Whether non-linear trends might better fit the data was beyond the scope of this study but might be a helpful approach for future hypothesis-driven research.

### Comparison with other national and international studies

Our finding of a significant increase in obesity among both men and women in Europe in the past two decades is in line with other trend studies [46–48]. A cross-country comparative study showing that the largest increase in Europe occurred in the United Kingdom also aligns with our finding of the largest increase in obesity in Scotland over this time period [47]. Cross-country comparative trend studies on socioeconomic inequalities in obesity are scarce. A comparison of our findings with trend studies in single countries (France, Norway), however, confirmed the increase in the prevalence of obesity in lower and higher educated groups, although not unanimously [18, 21]. One Swiss study reported a decline in absolute inequalities, due to a faster increase in the prevalence of obesity among higher educated [15]. The significant increase in absolute inequalities in Denmark among men is remarkable, especially as trends in social inequality for Denmark have not been reported before.

### Interpretation

Obesity increased particularly in Scotland and in Scandinavia (in Norway among men, and in Denmark among women). For Scotland, reports suggested that societal changes and technological developments have created an obesogenic environment over time that impedes to maintain healthy weight [49, 50]. Nordic countries are changing towards a less regulated market-liberalism potentially leading to an increased risk in obesity by a rise in fast-food industry in combination with a more stressful life [51]. While our findings on Scotland are in line with other studies, we were not able to find evidence why Scotland fared worse in the obesity epidemic. Indeed, major determinants of the change in obesity over time must be more prominent in Scotland than in other countries, but evidence which determinants these are is absent. For that reason we recommend to further expand cross-national comparative research on drivers of the obesity epidemic.

Apparently, the increase in obesity in almost all countries was not exclusive for either higher or lower socioeconomic groups. This finding further supports the notion that key determinants of obesity operate globally and affect entire populations. To the extent that the increase was larger for lower socioeconomic groups, the explanation must be that they were either more exposed to the underlying key determinants of obesity, or more vulnerable for such exposure. The rise in the prevalence has been attributed to societal and environmental changes resulting in obesogenic environments. Whereas initially differential exposure to obesogenic environment was thought to contribute to socioeconomic inequalities in obesity, Macintyre challenged the idea that lower socio-economic groups are more exposed to a food environment in which healthy food is less available and only at higher costs [52], a finding which has been empirically supported [53, 54]. Recent studies increasingly point towards social and cultural environments to explain the spread of obesity [55]. Such explanations for example suggest differences in cultural capital whereby healthy behaviors are used for the purpose of social distinction [56]. Whether and why lower socioeconomic groups are more vulnerable to obesogenic environments remains speculative.

One potential explanation is that resource scarcity (such as financial hardship) captures part of individuals’ cognitive capacity – referred to as ‘bandwidth’ - in a way that it impedes health behavior and as such may interfere with decision making, long-term planning and increase risk taking” [57, 58].

Whereas absolute inequalities in obesity widened, no increase in relative inequalities was found. This finding must be interpreted against the background of the overall increase in obesity; in a situation of increasing prevalence rates, it requires a substantially larger increase in the lower than the higher socioeconomic groups to achieve a widening in relative inequalities.

As such, the patterns described in this study for obesity deviate markedly from those seen in smoking. Whereas the prevalence of smoking decreased in the past years, and because the decrease in smoking was more pronounced among higher socioeconomic groups, absolute inequalities tended to decline (particularly among men) and relative inequalities increased. According to the theory of fundamental causes of Link and Phelan [59], socioeconomic inequalities persist over time due to the persistence of inequalities in access to socioeconomic resources and due to the fact that elimination of one determinant will only result in its replacement by another factor. Translating this theory to our example, obesity may be in the process of taking over part of the role of smoking as a determinant of inequalities in health.

### Implications

From a policy perspective, increasing prevalence rates and widening absolute inequalities are probably more relevant than widening relative inequalities. Thus, our findings further emphasizes the need for effective policies and interventions, with at least equal, but preferably larger effects among lower socioeconomic groups. For this purpose, Backholer et al. suggested that structural prevention strategies are likely to be more effective than approaches focusing on individual agents only, in which individuals are required to make independent choices [60]. Based on Backholer’s framework, Olstad et al. systematically analyzed a more comprehensive set of obesity prevention policies. They suggested a rather neutral impact of the majority of the reviewed interventions in the agento-structural continuum on inequalities in obesity. However, in line with Backholer they confirmed that structural policies implemented at the macroenvironmental level might be more likely to positively impact inequalities [61]. Recent evidence on the impact of a tax on sugar-sweetened beverage, for example, is encouraging in terms of the reduction of the prevalence of obesity in general, and requires further evaluation with regard to its equity impact [62, 63].

## Conclusions

The prevalence of obesity in European countries included in our study increased between 1990 and 2010, and more so in lower as compared to higher educational groups. As a result, absolute inequalities significantly increased between 1990 and 2010, whereas relative inequalities persisted or slightly declined. Our findings urge for a further understanding of the drivers of the increase in obesity in lower education groups, and an equity perspective in population-based obesity prevention strategies.

## Additional files

Additional file 1: Contains the results for trends in obesity prevalence for the low- and high-educated groups separately. The analysis was performed according to the method section described in the main manuscript. (PDF 219 kb)

Additional file 2: Contains the results for trends in obesity prevalence and occupation-related inequalities in obesity for both men and women. All analyses were performed according to the method section described in the main manuscript. (PDF 332 kb)

Additional file 3: Contains the results for education-related trends in slope index of inequality and relative index of inequality for obesity for both men and women. All analyses were performed according to the method section described in the main manuscript. (PDF 224 kb)

## Abbreviations

BMI: Body mass index
CI: Confidence interval
EU: European union
GDP: Gross domestic product
ISCED: International standard classification of education
OECD: Organization for economic co-operation and development
RD: Rate difference
RR: Rate ratio
