# Supplementary material for: Trends in educational inequalities in obesity in 15 European countries between 1990 and 2010
Source: Int J Behav Nutr Phys Act. 2017 May 8;14:63. doi: 10.1186/s12966-017-0517-8 (PMC5421333; doi:10.1186/s12966-017-0517-8)
Supplement: Supplementary file 1 — Contains the results for trends in obesity prevalence for the low- and high-educated groups separately. The analysis was performed according to the method section described in the main manuscript. (PDF 219 kb) [file 12966_2017_517_MOESM1_ESM.pdf]

Trends in educational inequalities in obesity in 15 European countries between 1990 and 2010

Kristina Hoffmann[1,2], Rianne De Gelder[1], Yannan Hu[1], Matthias Bopp[3], Jozsef Vitrai[4], Eero Lahelma[5], Gwenn Menvielle[6], Paula Santana[7], Enrique Regidor[8], Ola Ekholm[9], Johan P. Mackenbach[1], Frank J. van Lenthe[1]

[1]Department of Public Health, Erasmus MC, University Medical Center Rotterdam, The Netherlands

[2]Mannheim Institute of Public Health, Social and Preventive Medicine, Medical Faculty Mannheim, Heidelberg University, Mannheim, Germany

[3]Epidemiology, Biostatistics and Prevention Institute, University of Zürich, Switzerland

[4]National Institute for Health Development, Budapest, Hungary

[5]Department of Public Health, University of Helsinki, Helsinki, Finland

[6]Sorbonne Universités, INSERM, Institut Pierre Louis d'Epidémiologie et de Santé Publique (IPLESP UMRS 1136), Paris, France

[7]Departamento de Geografia, Centro de Estudos de Geografia e de Ordenamento do Territorio (CEGOT), Colégio de S. Jerónimo, Universidade de Coimbra, Coimbra, Portugal

[8]Department of Preventive Medicine and Public Health, Universidad Complutense de Madrid, Madrid, Spain

[9]National Institute of Public Health, Copenhagen, Denmark

Supplement 1

Supplement 1 contains the results for trends in obesity prevalence for the low- and high-educated groups separately. The analysis was performed according to the method section described in the main manuscript.

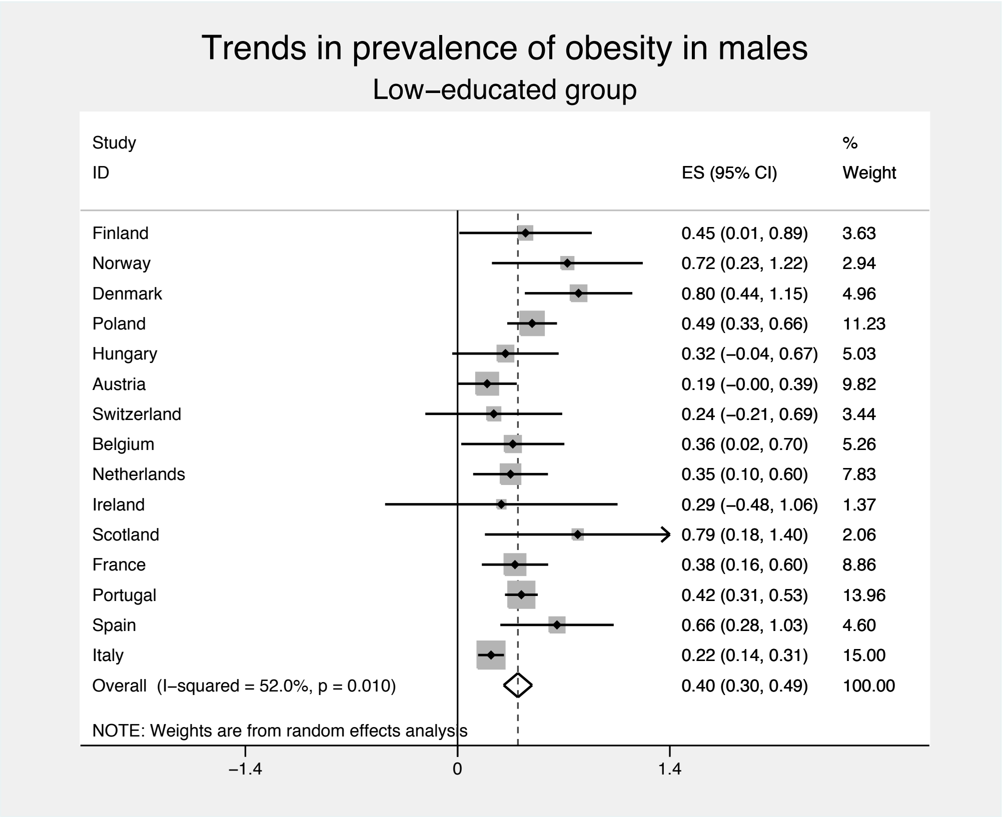

Figure S1a: Forest plot of meta-regression slopes for trends in prevalence of obesity (BMI ≥30 kg/m^2) in low-educated men. ES, effect estimator (% points change of obesity prevalence per year); CI, confidence interval.

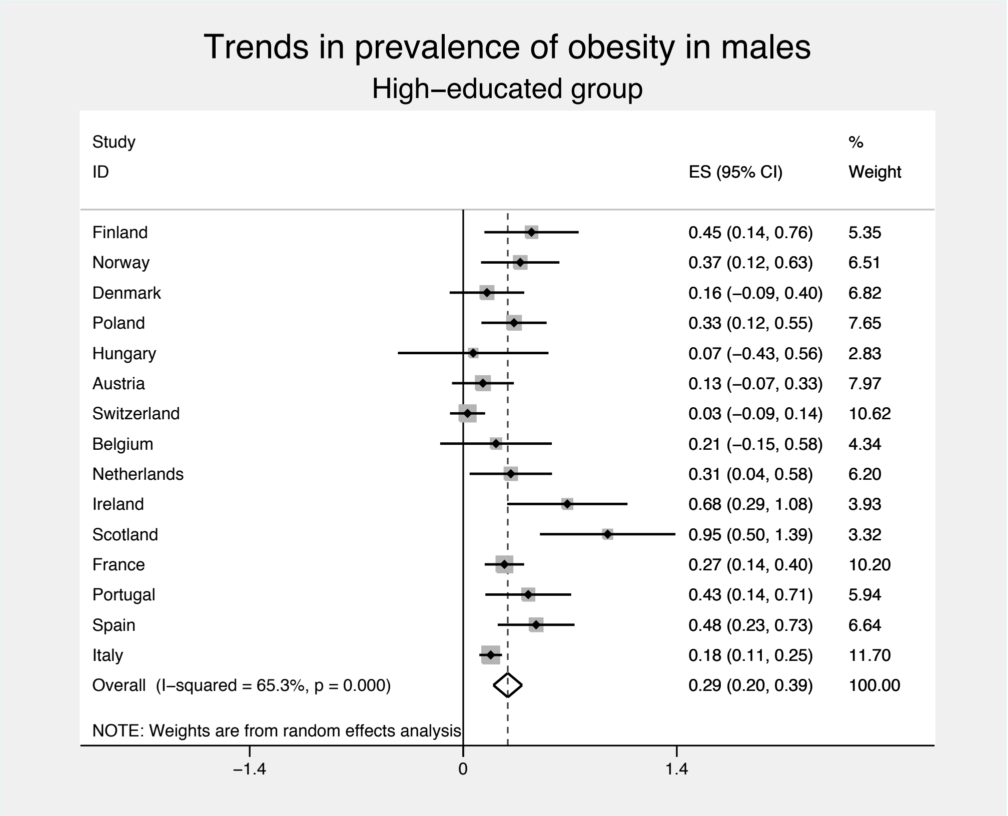

Figure S1b: Forest plot of meta-regression slopes for trends in prevalence of obesity (BMI ≥30 kg/m^2) in high-educated men. ES, effect estimator (% points change of obesity prevalence per year); CI, confidence interval.

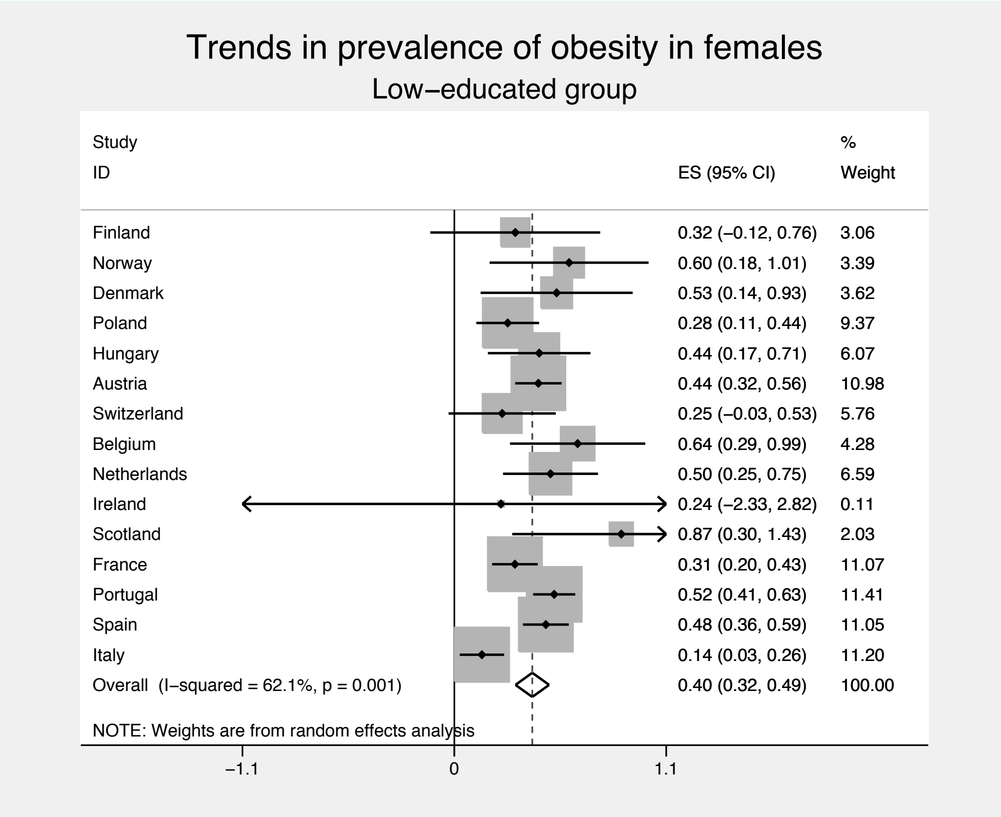

Figure S1c: Forest plot of meta-regression slopes for trends in prevalence of obesity (BMI ≥30 kg/m^2) in low-educated women. ES, effect estimator (% points change of obesity prevalence per year); CI, confidence interval.

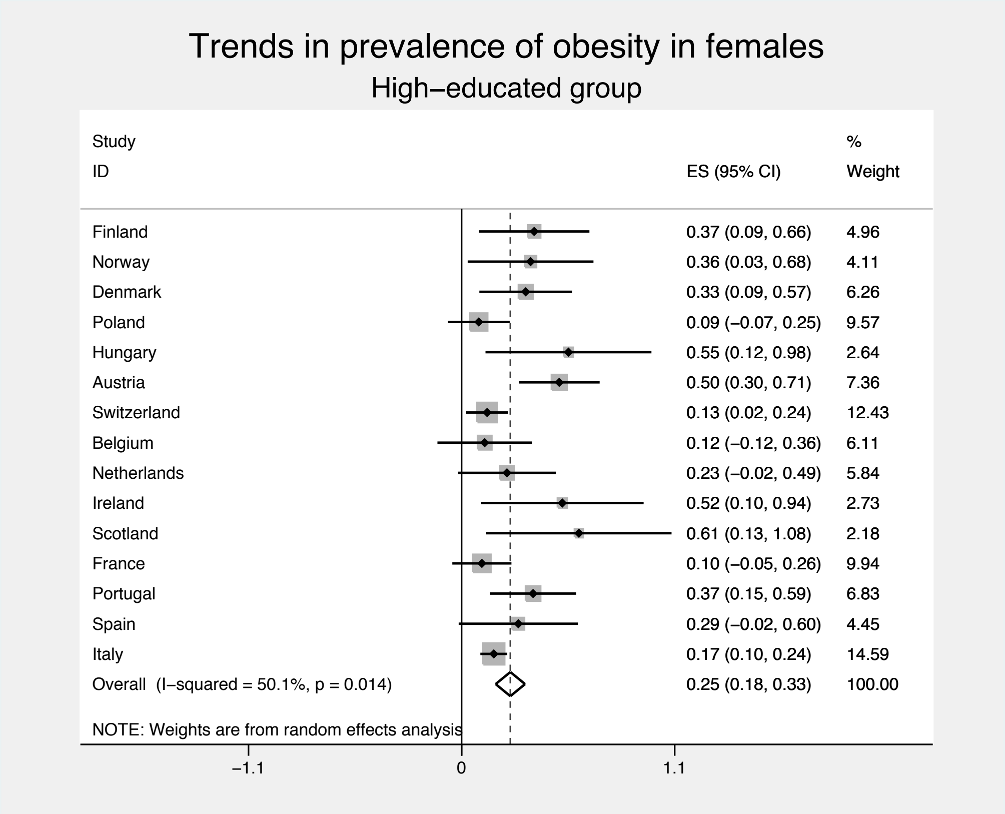

Figure S1d: Forest plot of meta-regression slopes for trends in prevalence of obesity (BMI ≥30 kg/m^2) in high-educated women. ES, effect estimator (% points change of obesity prevalence per year); CI, confidence interval.
