# Supplementary material for: Trends in educational inequalities in obesity in 15 European countries between 1990 and 2010
Source: Int J Behav Nutr Phys Act. 2017 May 8;14:63. doi: 10.1186/s12966-017-0517-8 (PMC5421333; doi:10.1186/s12966-017-0517-8)
Supplement: Supplementary file 2 — Contains the results for trends in obesity prevalence and occupation-related inequalities in obesity for both men and women. All analyses were performed according to the method section described in the main manuscript. (PDF 332 kb) [file 12966_2017_517_MOESM2_ESM.pdf]

Trends in educational inequalities in obesity in 15 European countries between 1990 and 2010

Kristina Hoffmann[1,2], Rianne De Gelder[1], Yannan Hu[1], Matthias Bopp[3], Jozsef Vitrai[4], Eero Lahelma[5], Gwenn Menvielle[6], Paula Santana[7], Enrique Regidor[8], Ola Ekholm[9], Johan P. Mackenbach[1], Frank J. van Lenthe[1]

[1]Department of Public Health, Erasmus MC, University Medical Center Rotterdam, The Netherlands

[2]Mannheim Institute of Public Health, Social and Preventive Medicine, Medical Faculty Mannheim, Heidelberg University, Mannheim, Germany

[3]Epidemiology, Biostatistics and Prevention Institute, University of Zürich, Switzerland

[4]National Institute for Health Development, Budapest, Hungary

[5]Department of Public Health, University of Helsinki, Helsinki, Finland

[6]Sorbonne Universités, INSERM, Institut Pierre Louis d'Epidémiologie et de Santé Publique (IPLESP UMRS 1136), Paris, France

[7]Departamento de Geografia, Centro de Estudos de Geografia e de Ordenamento do Territorio (CEGOT), Colégio de S. Jerónimo, Universidade de Coimbra, Coimbra, Portugal

[8]Department of Preventive Medicine and Public Health, Universidad Complutense de Madrid, Madrid, Spain

[9]National Institute of Public Health, Copenhagen, Denmark

Supplement 2

Supplement 2 contains the results for trends in obesity prevalence and occupation-related inequalities in obesity for both men and women. Occupational classes were categorized as "manual" (considered the lower level) versus "non-manual". A third category "self-employed" was omitted from analysis because it has not been distinguished between the kind of self-employed occupation and considered not to be informative on the socio-economic status of this group. Respondents who were not economically active, and who couldn't be classified on the basis of their own last occupation, were classified as missing. All analyses were performed according to the method section described in the main manuscript.

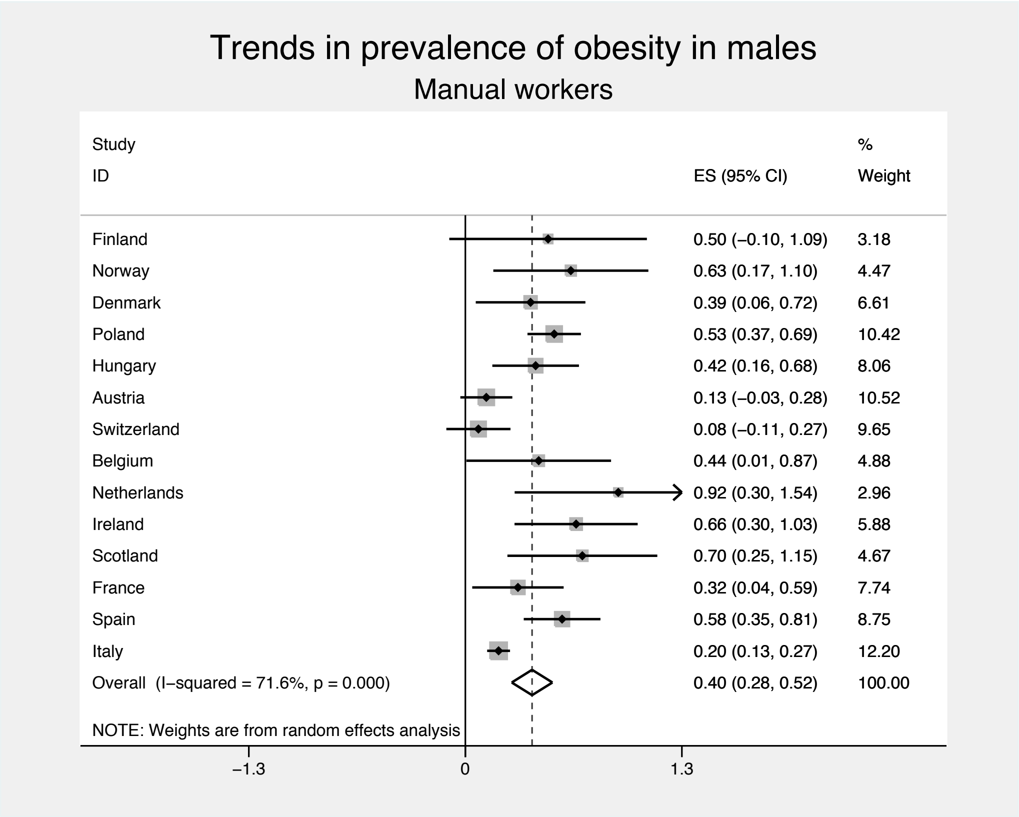

Figure S2a: Forest plot of meta-regression slopes for trends in prevalence of obesity (BMI ≥30 kg/m^2) in male manual workers. ES, effect estimator (% points change of obesity prevalence per year); CI, confidence interval.

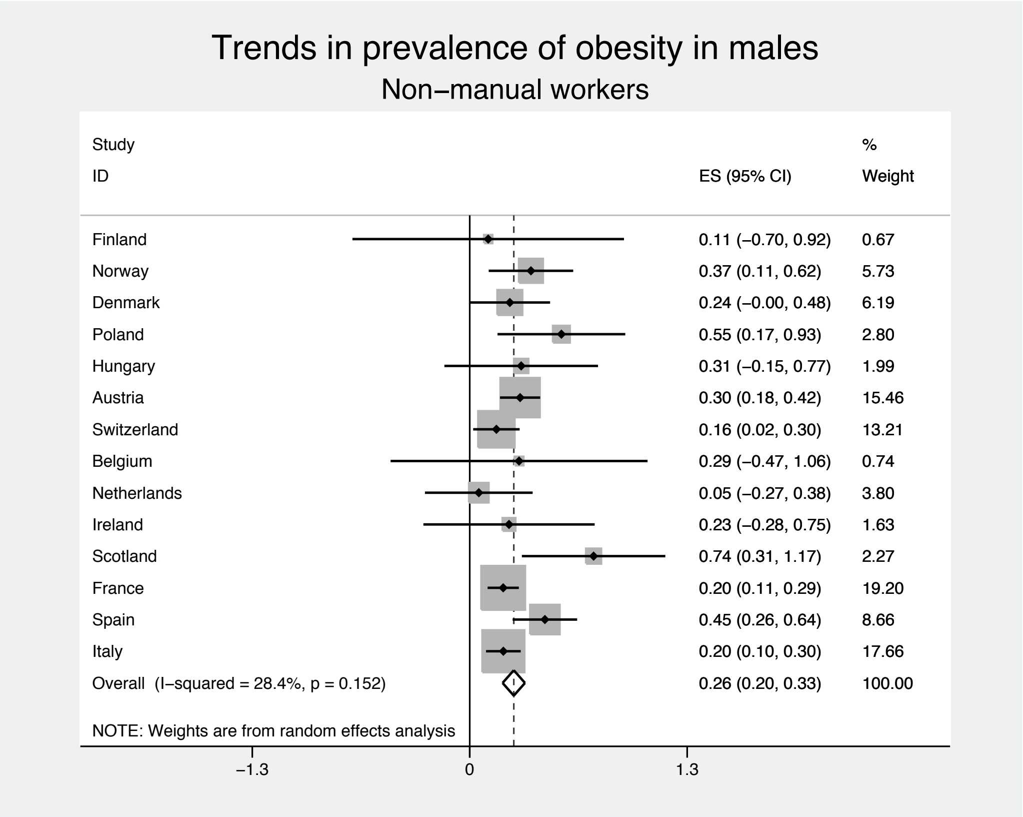

Figure S2b: Forest plot of meta-regression slopes for trends in prevalence of obesity (BMI ≥30 kg/m^2) in male non-manual workers. ES, effect estimator (% points change of obesity prevalence per year); CI, confidence interval.

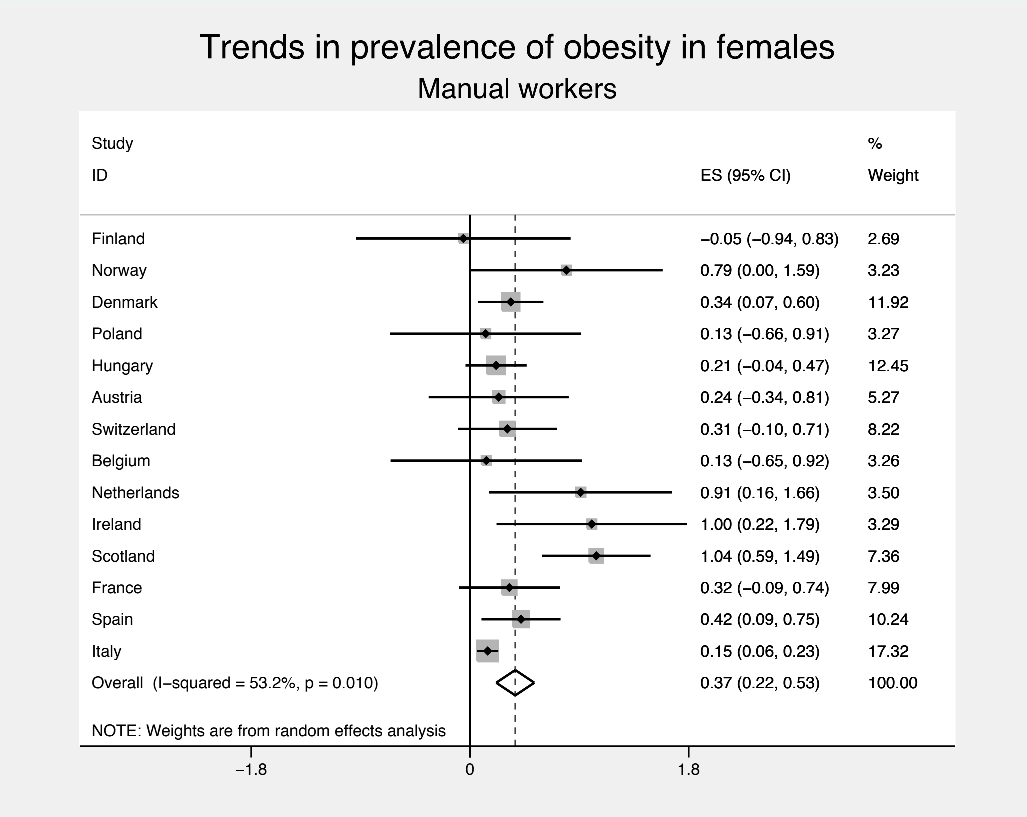

Figure S2c: Forest plot of meta-regression slopes for trends in prevalence of obesity (BMI ≥30 kg/m^2) in female manual workers. ES, effect estimator (% points change of obesity prevalence per year); CI, confidence interval.

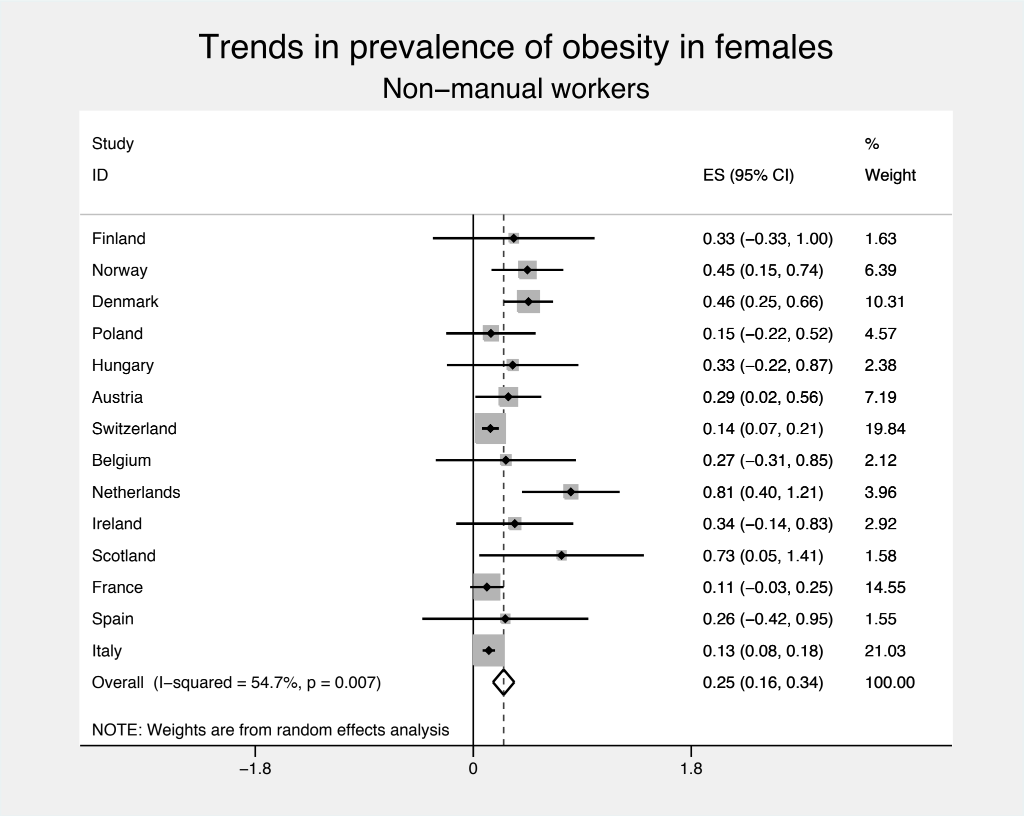

Figure S2d: Forest plot of meta-regression slopes for trends in prevalence of obesity (BMI ≥30 kg/m^2) in female non-manual workers. ES, effect estimator (% points change of obesity prevalence per year); CI, confidence interval.

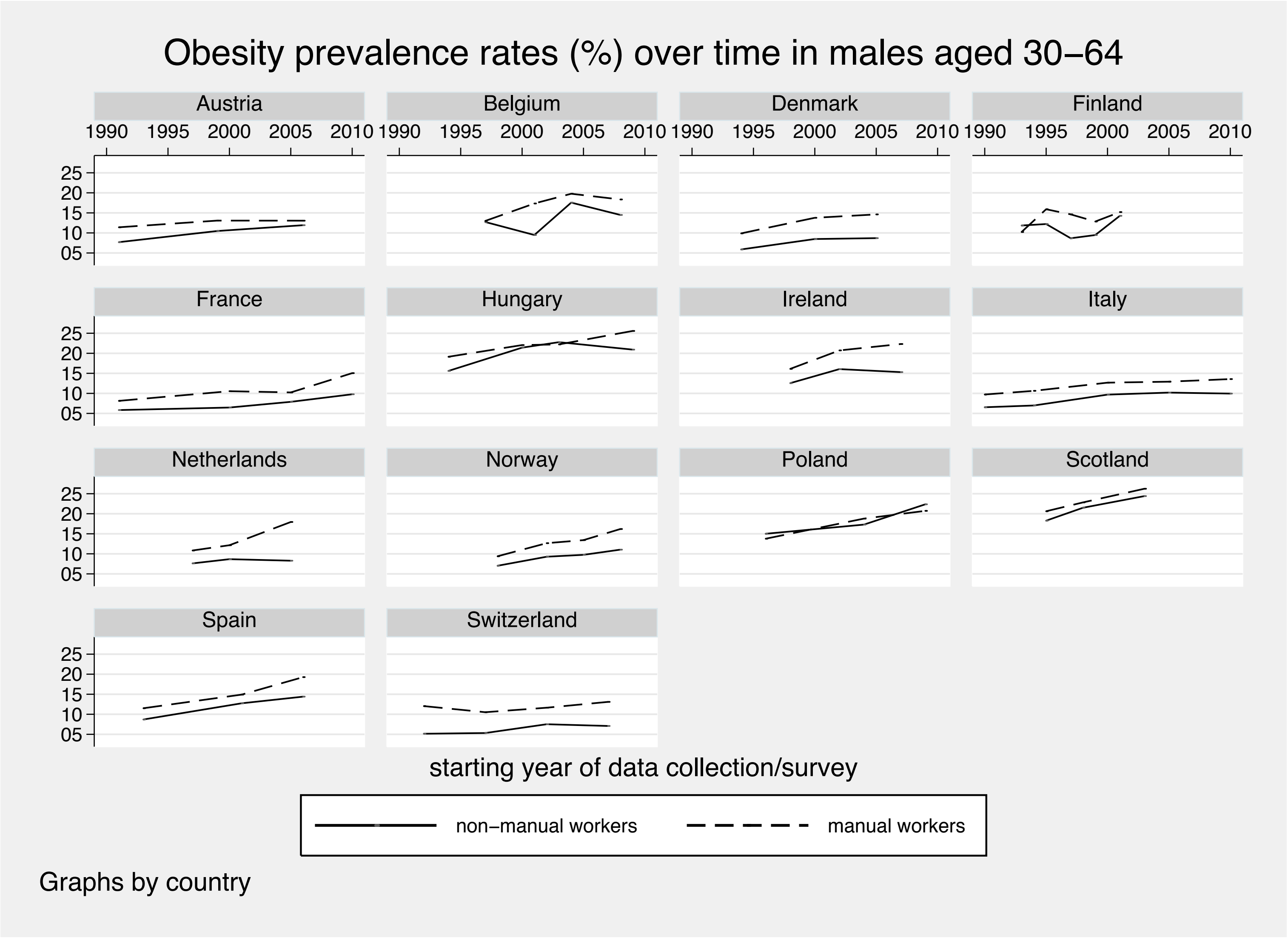

Figure S3a: Prevalence (%) of obesity (BMI ≥30 kg/m^2) over time for men aged 30-64 in all countries stratified by occupation (manual workers and non-manual workers) and total prevalence of obesity.

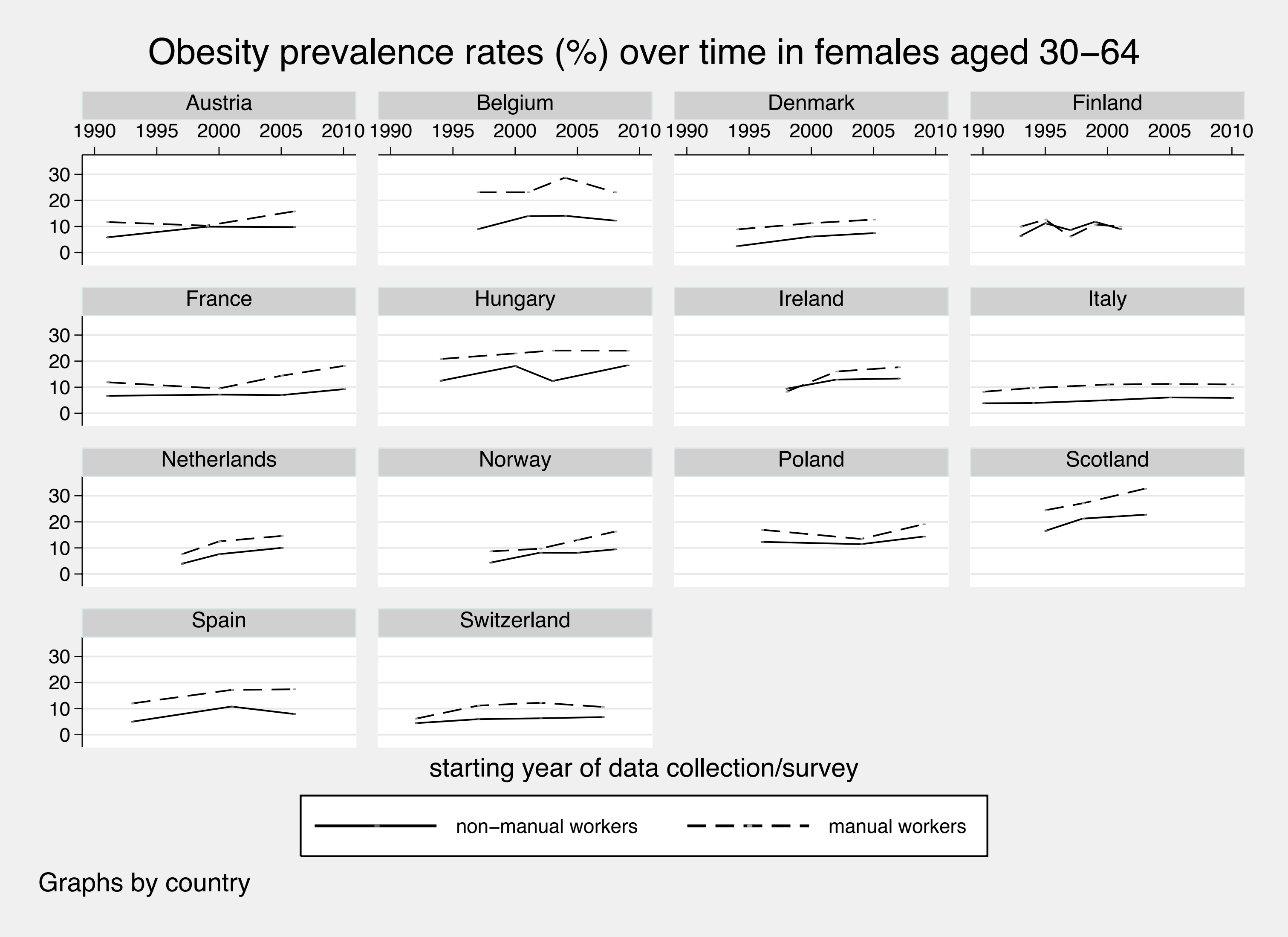

Figure S3b: Prevalence (%) of obesity (BMI ≥30 kg/m^2) over time for women aged 30-64 in all countries stratified by occupation (manual workers and non-manual workers) and total prevalence of obesity

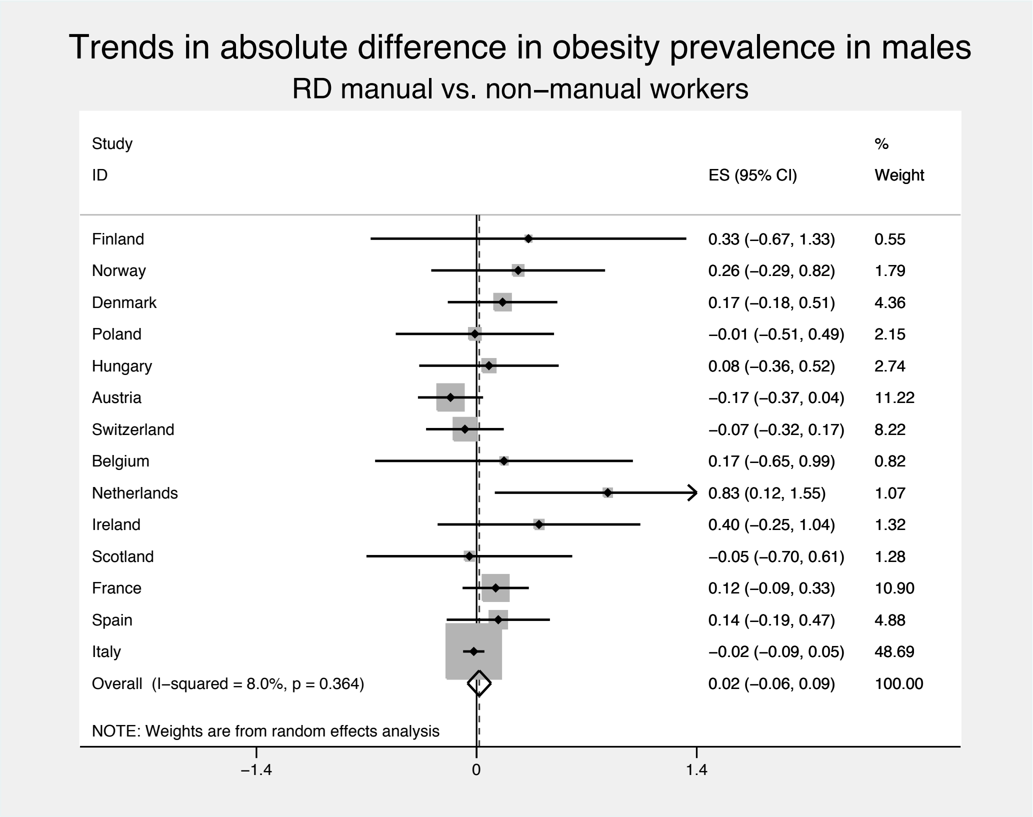

Figure S4a: Forest plot of meta-regression slopes for trends in absolute inequality in obesity (BMI ≥30 kg/m^2) in men. RD, rate difference; ES, effect estimator (% points change of obesity prevalence per year); CI, confidence interval.

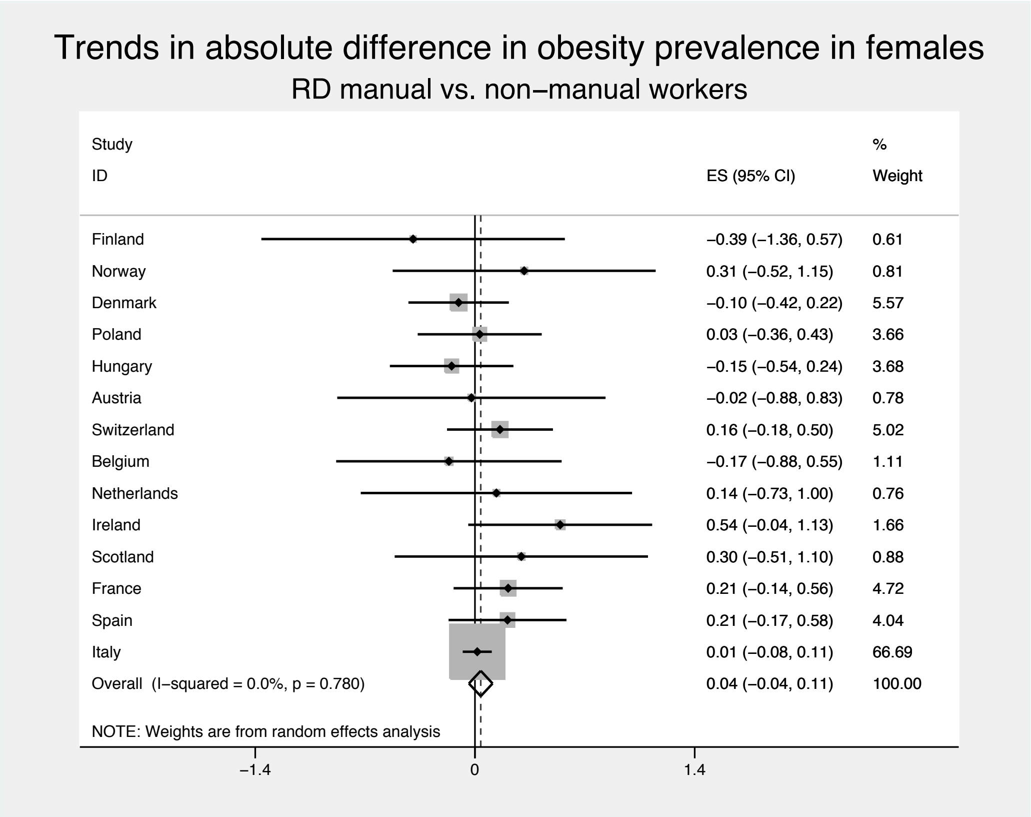

Figure S4b: Forest plot of meta-regression slopes for trends in absolute inequality in obesity (BMI ≥30 kg/m^2) in women. RD, rate difference; ES, effect estimator (% points change of obesity prevalence per year); CI, confidence interval.

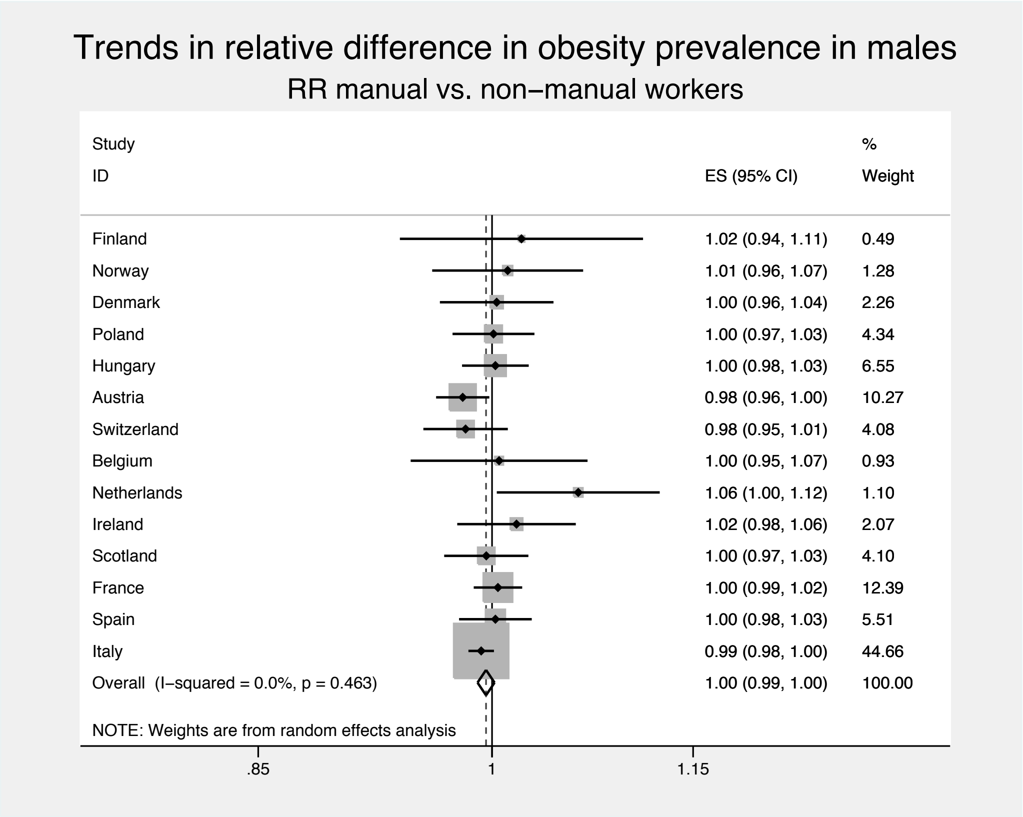

Figure S5a: Forest plot of meta-regression slopes for trends in relative inequality in obesity (BMI ≥30 kg/m^2) in men. RR, rate ratio; ES, effect estimator (relative change of relative inequalities in obesity per year); CI, confidence interval.

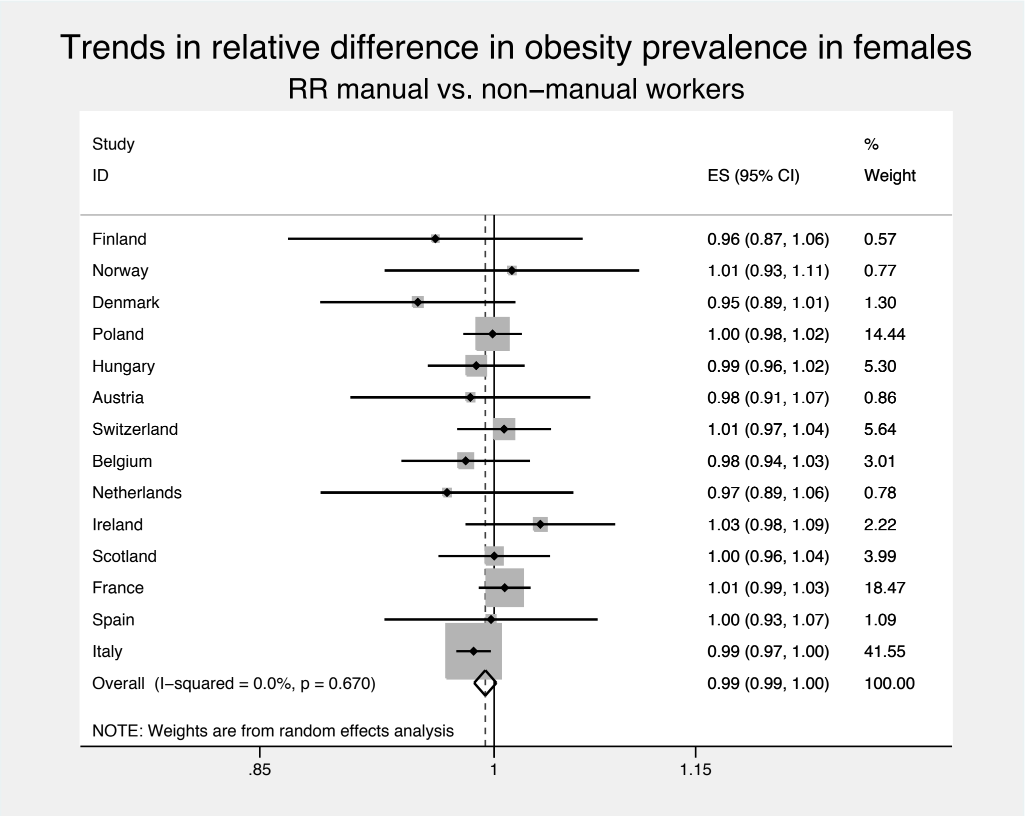

Figure S5b: Forest plot of meta-regression slopes for trends in relative inequality in obesity (BMI ≥30 kg/m^2) in women. RR, rate ratio; ES, effect estimator (relative change of relative inequalities in obesity per year); CI, confidence interval.
