# Supplementary material for: Trends in educational inequalities in obesity in 15 European countries between 1990 and 2010
Source: Int J Behav Nutr Phys Act. 2017 May 8;14:63. doi: 10.1186/s12966-017-0517-8 (PMC5421333; doi:10.1186/s12966-017-0517-8)
Supplement: Supplementary file 3 — Contains the results for education-related trends in slope index of inequality and relative index of inequality for obesity for both men and women. All analyses were performed according to the method section described in the main manuscript. (PDF 224 kb) [file 12966_2017_517_MOESM3_ESM.pdf]

Trends in educational inequalities in obesity in 15 European countries between 1990 and 2010

Kristina Hoffmann[1,2], Rianne De Gelder[1], Yannan Hu[1], Matthias Bopp[3], Jozsef Vitrai[4], Eero Lahelma[5], Gwenn Menvielle[6], Paula Santana[7], Enrique Regidor[8], Ola Ekholm[9], Johan P. Mackenbach[1], Frank J. van Lenthe[1]

[1]Department of Public Health, Erasmus MC, University Medical Center Rotterdam, The Netherlands

[2]Mannheim Institute of Public Health, Social and Preventive Medicine, Medical Faculty Mannheim, Heidelberg University, Mannheim, Germany

[3]Epidemiology, Biostatistics and Prevention Institute, University of Zürich, Switzerland

[4]National Institute for Health Development, Budapest, Hungary

[5]Department of Public Health, University of Helsinki, Helsinki, Finland

[6]Sorbonne Universités, INSERM, Institut Pierre Louis d'Epidémiologie et de Santé Publique (IPLESP UMRS 1136), Paris, France

[7]Departamento de Geografia, Centro de Estudos de Geografia e de Ordenamento do Territorio (CEGOT), Colégio de S. Jerónimo, Universidade de Coimbra, Coimbra, Portugal

[8]Department of Preventive Medicine and Public Health, Universidad Complutense de Madrid, Madrid, Spain

[9]National Institute of Public Health, Copenhagen, Denmark

Supplement 3

Supplement 3 contains the results for education-related trends in slope index of inequality SII and relative index of inequality RII for obesity for both men and women. All analyses were performed according to the method section described in the main manuscript.

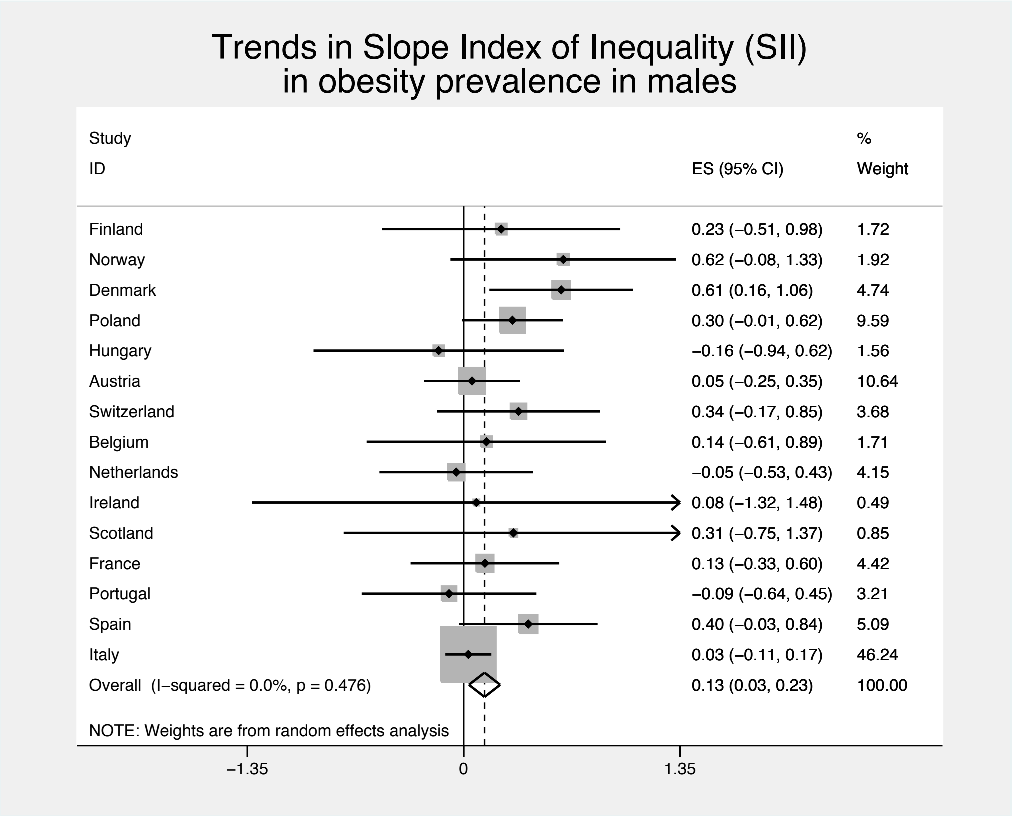

Figure S6a: Forest plot of meta-regression slopes for Slope Index of Inequality (SII) in prevalence of obesity (BMI ≥30 kg/m2) in men. ES, effect estimator (% points change of obesity prevalence per year); CI, confidence interval.

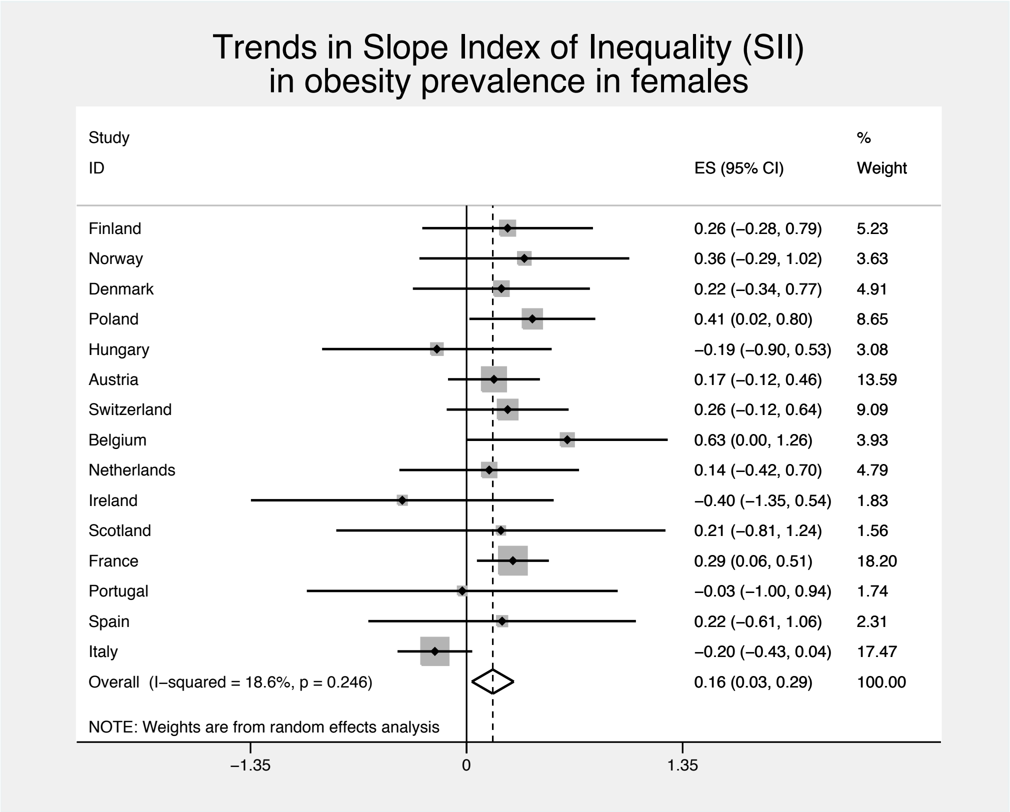

Figure S6b: Forest plot of meta-regression slopes for Slope Index of Inequality (SII) in prevalence of obesity (BMI ≥30 kg/m2) in women. ES, effect estimator (% points change of obesity prevalence per year); CI, confidence interval.

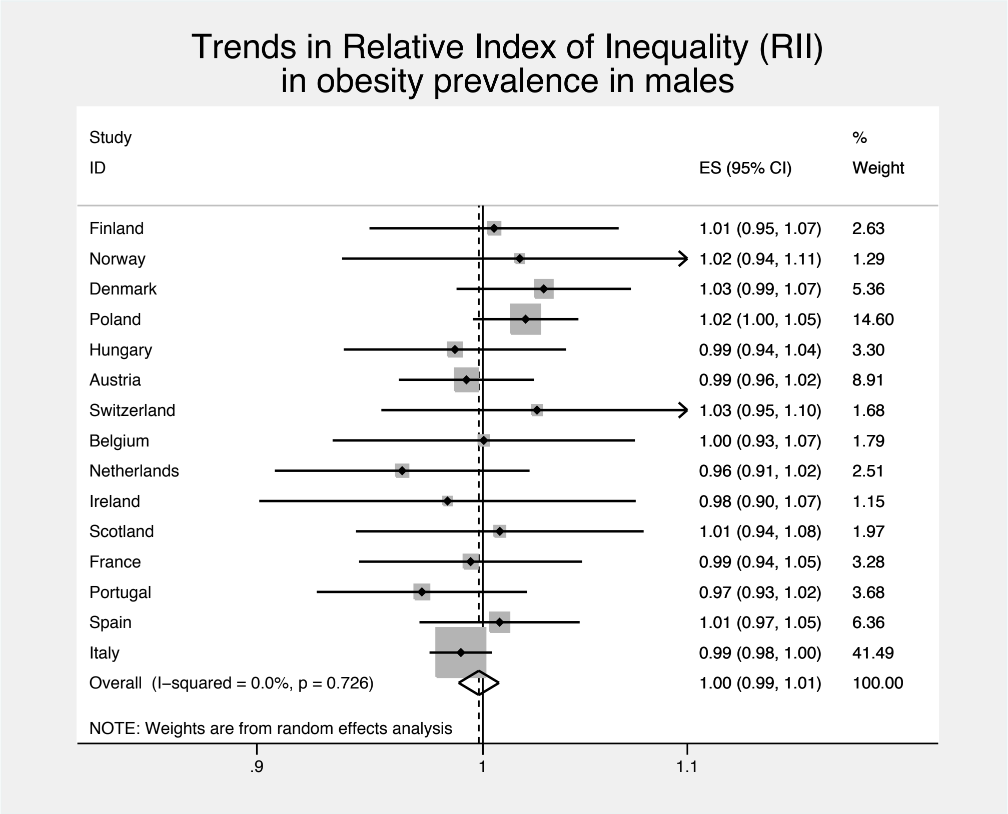

Figure S7a: Forest plot of meta-regression slopes for Relative Index of Inequality (RII) in prevalence of obesity (BMI ≥30 kg/m2) in men. ES, effect estimator (% points change of obesity prevalence per year); CI, confidence interval.

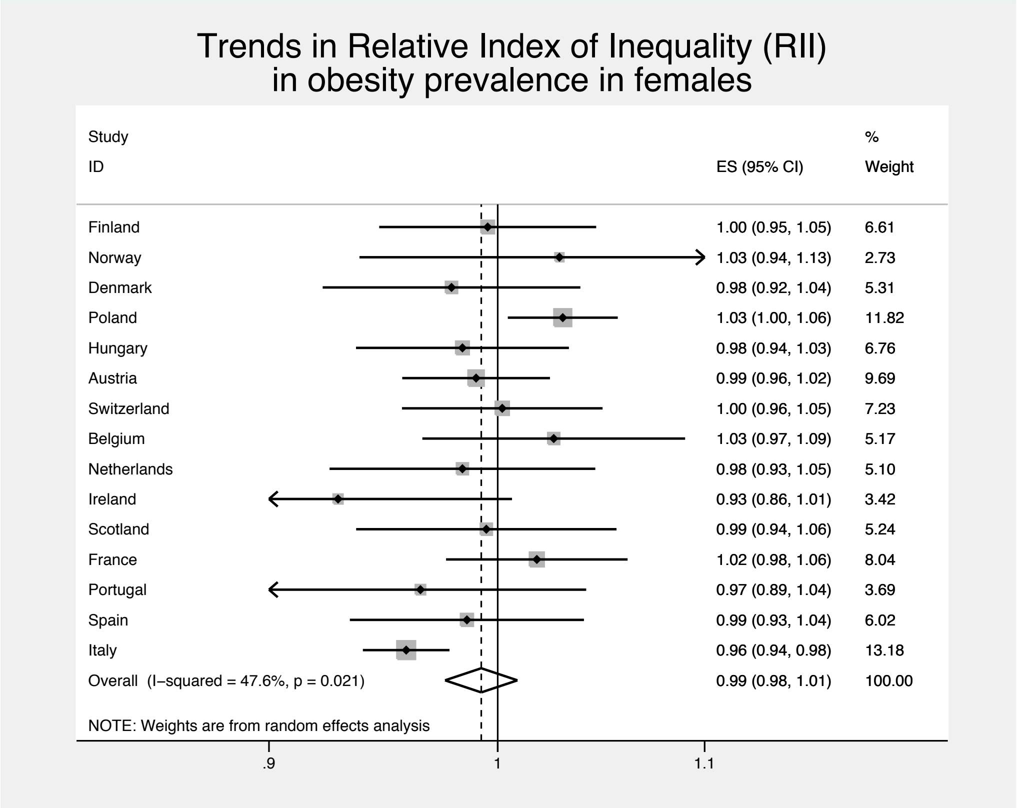

Figure S7b: Forest plot of meta-regression slopes for Relative Index of Inequality (RII) in prevalence of obesity (BMI ≥30 kg/m2) in women. ES, effect estimator (% points change of obesity prevalence per year); CI, confidence interval.
